# Stabilization of Tuberculosis Reporter Enzyme Fluorescence (REFtb) Diagnostic Reagents for Use at the Point of Care

**DOI:** 10.3390/diagnostics12071745

**Published:** 2022-07-19

**Authors:** Maxim Lebedev, Aaron B. Benjamin, Kent J. Koster, Kathryn E. Broyles, Sathish Kumar, Joseph M. Jilka, Jeffrey D. Cirillo

**Affiliations:** 1 Department of Microbial Pathogenesis and Immunology, Texas A&M School of Medicine, Bryan, TX 77807, USA; aaronbbenjamin@tamu.edu (A.B.B.); kkoster@tamu.edu (K.J.K.); katiebroy1@tamu.edu (K.E.B.); kskumar@tamu.edu (S.K.); 2 Pulmonescence Diagnostics, Inc., Temple, TX 76504, USA; jjilka@pulmondx.com

**Keywords:** tuberculosis, diagnostics, beta lactamase, BlaC, lactose, mannitol, Blue Sepharose, excipient, lyophilization

## Abstract

Tuberculosis is one of the most frequent causes of death in humans worldwide. One of the primary reasons tuberculosis remains a public health threat is that diagnosis can take weeks to months, is often not very sensitive and cannot be accomplished in many remote environments. A rapid, sensitive and inexpensive point-of-care (POC) diagnostic would have a major impact on tuberculosis eradication efforts. The tuberculosis diagnostic system REFtb is based on specific detection of the constitutively expressed β-lactamase (BlaC) in *Mycobacterium tuberculosis* using a custom fluorogenic substrate designated as CDG-3. REFtb has potential as a diagnostic for tuberculosis that could be very inexpensive (<USD 2.00/test), used at the POC and could provide definitive diagnosis within 10 min. However, the reagents for REFtb are currently in liquid form, making them more susceptible to degradation and difficult to transport. We evaluated the improvement in the stability of REFtb reagents by lyophilization under a variety of conditions through their effects on the performance of REFtb. We found that lyophilization of REFtb components produces an easily reconstituted powder that displays similar performance to the liquid system and that lactose represents one of the most promising excipients for use in a final POC REFtb diagnostic system. These studies provide the foundation for the production of a stable POC REFtb system that could be easily distributed worldwide with minimal or no requirement for refrigeration.

## 1. Introduction

Tuberculosis (TB) is a major global health problem. According to the World Health Organization (WHO), an estimated 10 million people become infected (including 1.1 million children), and 1.5 million people die from this disease each year. Approximately 2 billion people (a quarter of the global population) are thought to be currently infected with *Mycobacterium tuberculosis* (Mtb). Drug-resistant TB is continuing to increase worldwide and poses a critical threat to public health, with death rates from 40 to 60% [1]. The WHO set a target to reduce the death rate from TB by 90% and incidence by 80% compared to 2015. In order to achieve this target, a fast, sensitive and low-cost point-of-care (POC) diagnostic system is crucial, but treatment advances will also likely be needed [1].

The gold standard bacteriological tests, such as culture and smear microscopy, require laboratory infrastructure and multiple visits to the clinic to confirm diagnosis [2,3,4,5]. Smear microscopy is rapid and low cost, but the low sensitivity prevents it from being optimal as a diagnostic tool [1,6,7]. Culturing Mtb in a diagnostic lab takes weeks to months, and its specificity is frequently impaired by contaminants [6,8,9,10]. Delayed diagnosis increases the risk of TB transmission, affecting morbidity and mortality rates and increasing the risk of drug resistance [11].

One of the approaches to improve TB diagnostics is the discovery of tuberculosis-associated biomarkers, but progress in this direction has been slow, and use of biomarkers in clinical TB diagnosis is often difficult to implement [12,13,14,15,16,17]. Nevertheless, several effective diagnostics, including GeneXpert MTB/RIF, have recently been developed and deployed [18]. GeneXpert provides the ability to detect Mtb with high sensitivity and test for single drug resistance in approximately 2 h [12,19], but the costs associated with GeneXpert are prohibitive for certain regions [2,19,20]. Another approach that allows fast results is the interferon-gamma release assay (IGRA), including the QuantiFERON^®^-TB Gold In-Tube test and T-SPOT^®^ TB test. Some disadvantages of this approach are the inability to differentiate latent and active forms of Mtb infection. Additionally, IGRA is unable to identify prior infection and displays high background from non-tuberculous mycobacterial infections [21,22].

A rapid, sensitive and inexpensive POC diagnostic test that allows the detection of Mtb in clinical samples, including sputum, urine or fecal diagnostic material, would dramatically improve the TB diagnostic landscape. We developed a novel diagnostic technology designated as reporter enzyme fluorescence (REF) [23,24,25,26], which is based on the detection of BlaC (Rv2068c), an endogenous β-lactamase constitutively expressed by Mtb [27,28]. BlaC is localized to the surface of the bacteria as well as secreted [23] and has extremely high catalytic activity [29,30]. The structure of the BlaC active site makes this enzyme unique among β-lactamases [31], allowing the development of specific substrates. We developed substrates that have the ability to detect 1–10 Mtb bacilli, even in the presence of >10^5^ other bacterial species expressing other β-lactamases [25,32,33]. Using the REFtb reagent system, the same specificity and sensitivity are observed for the Mtb BlaC within human clinical samples as those observed in laboratory buffers [26]. REFtb has a sensitivity of 88.1% and a specificity of 86.1% in suspected TB patients, demonstrating that it is more sensitive than smear microscopy [13,25,26]. Beyond these promising data in human sputum samples, the real strength of REFtb is that it is very fast (within 10 min) and inexpensive (<USD 2.00), allowing more expensive, high-specificity diagnostic tests, such as GeneXpert, to only be used when needed. The high sensitivity of REFtb would miss very few cases, a great improvement over existing case-finding strategies. In order for REFtb to have the greatest impact on preventing TB transmission, it must be available in all areas where TB is prevalent. Use at the POC will require shipping throughout the world, use by personnel that may have limited technical knowledge and simple portable battery-operated readers. Shipping throughout the world would be optimal if the reagents, including the substrate, have a long shelf-life at nearly any likely ambient temperature. As a preliminary step toward translating the REFtb test into a useful POC test, we investigated lyophilization strategies that could be used to ensure the stability and performance of the REFtb reagents.

Lyophilization or freeze drying is a process where water is removed from a frozen sample by sublimation of ice in a vacuum. The preservation and stabilization of numerous biologicals as well as small molecules in pharmaceutical and biotechnology industries are accomplished using specific lyophilization conditions [34,35]. The optimal lyophilization strategy will stabilize the molecular structure in the dry state, often ensuring longer shelf-life, even at temperatures higher than standard room temperature with conditioned air (24 °C). As part of the lyophilization process, inert additives, called excipients, are usually added to provide improved stability to the active molecules present and protect the active components from stress upon freezing and lyophilization. Excipients can serve as bulking agents, buffering agents and collapse temperature modifiers [34,36]. The main function of bulking excipients is to provide quality dried cake formation, characterized by the formation of pores that help the vapor escape from the product during the drying cycle as well as improved solubility as the cake is dissolved. Sugars are widely used as inert bulking excipients, of which the most widely used are mannitol and lactose [36]. We examined the impact of freeze drying on the activity, solubility, structural integrity and performance of key reagents for REFtb as well as how different excipients impact these parameters. These studies provide evidence that there is potential for the REFtb reagent system to be stabilized by lyophilization for shipping, which will provide sufficient shelf-life for it to be used as a POC diagnostic test for TB throughout the world.

## 2. Materials and Methods

CDG-3 is the key component of the REFtb diagnostic system. To determine suitable storage and shipping conditions for the substrate, we compared how liquid and lyophilized CDG-3 retain their stability. We also compared the stability of these two forms with the addition of three sugars as excipient candidates. We utilized two approaches to evaluate the substrate. The first approach was to measure the fluorescent signal of the product before and after the cleavage of the substrate by purified BlaC, while the second was to analyze it using high-performance liquid chromatography (HPLC) in comparison with fresh untreated substrate as a control.

### 2.1. Testing the Stability of Lyophilized CDG-3

Using MES buffer (0.457 M, pH 6.0) as a diluent, the following four solutions were prepared to a final concentration of 48.7 mg/mL: 1—MES buffer alone (Omni Pur, Millipore, Caldwell, ID, USA), 2—mannitol (VWR, Radnor, PA, USA), 3—α-lactose (Sigma, Kawasaki, Japan) and 4—D-raffinose (Research Products International, Mt Prospect, IL, USA). An amount of 10 mM CDG-3 (Acme Bioscience, Inc., Palo Alto, CA, USA) in dimethyl sulfoxide (DMSO) was added to produce combinations 1–4 at a ratio of 1:2000. Each CDG-3 + excipient combination was analyzed using HPLC and tested for threshold of detection using purified BlaC (see below). After reaction with BlaC, BlaC-treated substrate, the samples were also analyzed via HPLC (from the samples with the highest concentration of BlaC).

The rest of each combination was split into five equal parts (taken in duplicate). Each part was assigned to the following experimental conditions: 1—non-lyophilized, exposed to room temperature (RT) for 24 h; 2—non-lyophilized, exposed to 60 °C for 24 h; 3—exposed to RT for 24 h in lyophilized form; 4—exposed to 60 °C for 24 h in lyophilized form; 5—lyophilized, but unexposed to any temperatures (immediately reconstituted and tested). Two samples of 0.6 mL and two samples of 0.9 mL were taken.

After 24 h of incubation at RT or 60 °C, CDG-3 lyophilized forms were reconstituted in sterile deionized (DI) water (the amount of water for reconstitution was determined based on the actual loss of water upon lyophilization). The samples were tested with a series of BlaC concentrations and analyzed using HPLC (see protocol below). Product fluorescence was compared across samples with these excipients and without excipient for each experimental condition.

The reaction of BlaC with CDG-3 substrate without exposure to high temperatures was used as a point of reference (as a zero-time point) for the comparison of the reaction results after exposing the substrate to room temperature and 60 °C in lyophilized vs. non-lyophilized (liquid) forms.

### 2.2. Evaluating the Effect of Lyophilization on Blue Sepharose

Blue Sepharose-6 Fast Flow (Cytiva) stock solution was centrifuged (swing bucket, 4000 rpm, 10 min). The supernatant was removed, and the pellet was resuspended in MES buffer (0.914 M, pH 6.0) in the same volume as the original stock Blue Sepharose and centrifuged again. After centrifugation, the supernatant was removed, and the pellet was resuspended in the MES buffer again.

Blue Sepharose in MES was placed at −80 °C for 3 h (vials were weighed before freezing), then lyophilized at 0.05 mBar, −48 °C (collector) overnight. After lyophilization powder was reconstituted in the volume of sterile DI water that was removed by lyophilization, fresh Blue Sepharose was prepared using the same protocol.

An amount of 500 µg/mL bovine serum albumin (BSA) (Thermo Scientific, Waltham, MA, USA) was prepared in the MES buffer pH6. The resulting solution was tested using a Pierce bicinchoninic acid (BCA) protein assay kit (Thermo Scientific) according to the manufacturer’s protocol.

Lyophilized and non-lyophilized Blue Sepharose was distributed to 0.5 mL portions and centrifuged at 8000 rpm for 5 min. The supernatant was removed, and pellets were re-suspended in 1 mL of MES buffer with 500 µg/mL of albumin. Each sample was transferred to a 24-well plate, sealed and incubated on a shaker at RT for 1 h (first cycle of treatment).

After incubation, samples were centrifuged, and the supernatant was tested using the BCA protein test and mixed with the next portion of Blue Sepharose (pelleted by centrifugation). Each sample was transferred to a 24-well plate, sealed and incubated on a shaker at 25 °C for 1 h (second cycle of treatment). The same procedure was used for the third, fourth and fifth cycles of treatment with Blue Sepharose. After each cycle of treatment, a sample of each experimental specimen was collected for the BCA protein test. The BCA protein assay was performed in triplicate for each sample using the manufacturer’s instructions.

The resulting samples of MES with depleted BSA after the fifth treatment cycle were tested for the thresholds of detection of BlaC using REFtb assays in triplicate.

### 2.3. Testing the Effect of Temperature on Lyophilized Blue Sepharose

Blue Sepharose stock solution was centrifuged (swing bucket, 4000 rpm, 10 min), the supernatant was removed, and the pellet of Blue Sepharose was resuspended in the same volume of MES buffer (0.914 M, pH 6.0) as the Blue Sepharose stock suspension and centrifuged again. The supernatant was removed, and Blue Sepharose was resuspended in the same volume of MES buffer as before.

The resulting Blue Sepharose suspension was dispensed into 8 vials, 0.5 mL per vial, and frozen at −80 °C. The frozen Blue Sepharose was lyophilized at 0.05 mBar, −48 °C (collector) overnight. Leftover non-lyophilized suspended Blue Sepharose was stored in the refrigerator overnight and used as non-lyophilized experimental samples.

Two vials of lyophilized Blue Sepharose and two vials of non-lyophilized suspension were incubated at RT or 60 °C for 24 h. The control samples of fresh Blue Sepharose (not lyophilized and not exposed to temperature (control) were kept in the refrigerator. After incubation, the lyophilized Blue Sepharose was reconstituted in the exact volume of sterile DI water that was removed by lyophilization.

An amount of 500 µg/mL of BSA solution was produced in the MES buffer pH6 and used to resuspend all the experimental samples of Blue Sepharose pellets created by centrifugation. Each sample was transferred to a 24-well plate and incubated on a shaker at RT for 1 h, sealed. After incubation, samples were centrifuged, and the supernatant was tested using the BCA protein test and BlaC-CDG enzymatic reaction (described below) for thresholds of detection.

### 2.4. HPLC Analysis

The following procedure was used for all HPLC analyses described in this work. An amount of 60 µL of the sample was mixed with 6 µL of trifluoroacetic acid (TFA) (VWR Chemicals), mixed with pipetting. (50 µL = 1 µmol). Samples were injected to HPLC (Waters e2695 with 2998 PDA detector) onto a Luna Omega 3 uM Polar C18 100 A column using a gradient of 0–15% H_2_O + 0.1%TFA:ACN + 0.1%TFA over 15 min for each sample.

To monitor the substrate and product, the following wavelengths were used: 215 nm, 254 nm, 280 nm and 190–800 nm. The results were analyzed at 215 nm.

### 2.5. Test for the Threshold of Detection

Six serial dilutions (5-fold) of purified recombinant BlaC (50.5 uM) in a MES buffer (97.5 mg/mL, pH to 6.0) were produced starting from 1:25 in total volume, enough for all 15 µL triplicate samples. An amount of 5 µL of diluted BlaC was added to appropriate wells of the 384-well black-wall plate (Nunc, Thermo Scientific) in triplicate for each sample up to the sixth well. The seventh and eighth wells were “CDG-3 only” (background) and “no substrate” controls, respectively. An amount of 5 µL of the appropriate reconstituted CDG-3 substrate was added to the first 7 wells of each sample (in triplicate).

Fluorescent scanning was performed with 492 nm excitation and 535 nm emission filters every 10 min for 1 h using TriStar LB 941 plate reader (Berthold Technologies, Bad Wildbad, Germany). Fluorescence of the cleavage product (fluorescence minus background) as well as Δ fluorescence (the difference between current product fluorescence and product fluorescence at zero-time point) were determined.

### 2.6. Data Analysis

MS Excel was used for data handling, calculations, creation of spreadsheets and graphs. Statistical analysis as a *T*-test and analysis of variance with post hoc Tukey HSD test were performed.

## 3. Results

CDG-3 substrate had never previously been lyophilized at working concentrations in an MES buffer, so the first question was the effect of this procedure itself on substrate stability and functionality, both with and without sugar excipients present. The second major question of this work was to test lyophilized CDG-3 stability with and without excipients at room temperature and 60 °C.

### 3.1. Lyophilization, Followed by Reconstitution, Slightly Reduced the Functional Activity of CDG-3 Substrate, but Lactose Helped Protect It from Degradation

**Figure 1:**
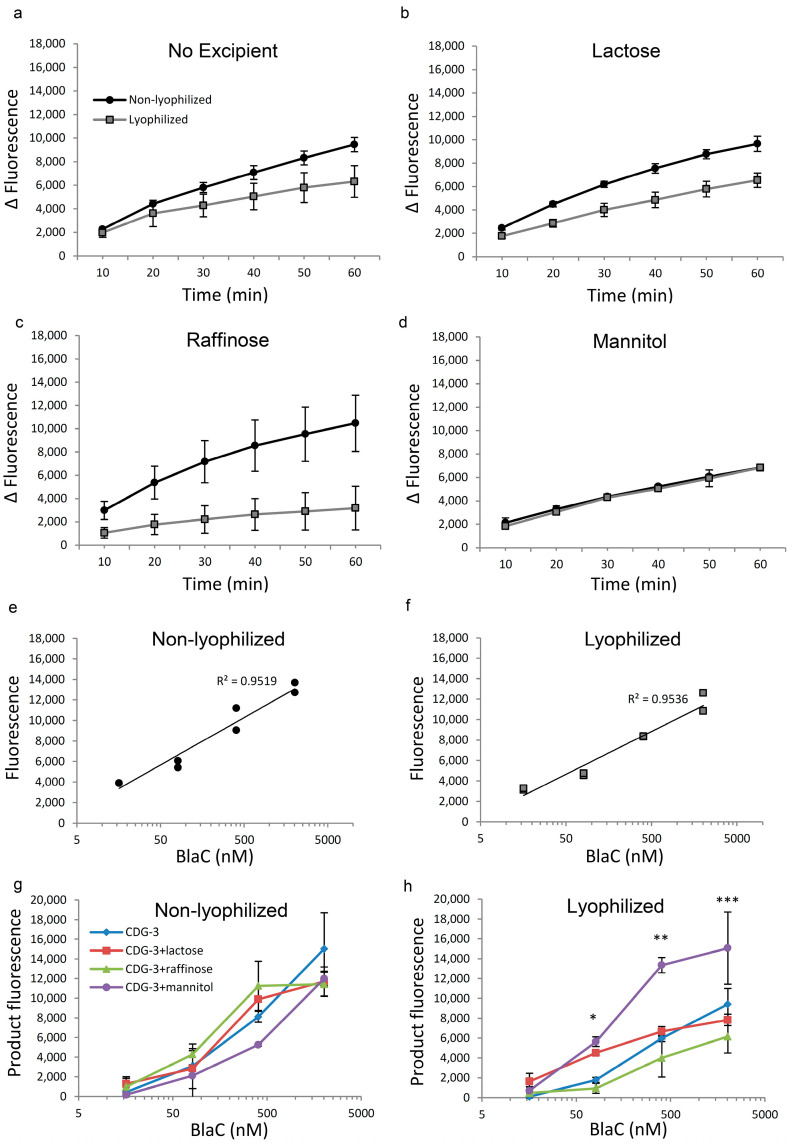
Excipients help stabilize the lyophilized CDG-3. Reaction of BlaC with non-lyophilized vs. lyophilized and reconstituted CDG-3 substrate without exposure to higher temperatures Fluorescence was used to compare the reaction rate of CDG-3 with BlaC in lyophilized vs. non-lyophilized samples (two experimental replicates per condition). (**a**)—Kinetics of the reaction of purified 404 nM BlaC and CDG-3 substrate without excipients; (**b**)—in the presence of lactose; (**c**)—in the presence of raffinose; (**d**)—in the presence of mannitol; (**e**)—Relationships of BlaC concentration with fluorescence in non-lyophilized and (**f**)—in lyophilized samples; (**g**)—Changes in fluorescence within 30 min of incubation with and without excipients in non-lyophilized and (**h**)—lyophilized samples. BlaC concentrations: 2022 nM, 404 nm, 81 nM and 16 nM. Error bars represent standard deviation. * *p* < 0.01 in the comparison between: CDG-3 and lactose, CDG-3 and mannitol, lactose and raffinose, lactose and mannitol; ** *p* < 0.01 in the comparison between: CDG-3 and mannitol, raffinose and mannitol, *p* < 0.05 in the comparison between lactose and mannitol; *** *p* < 0.05 between raffinose and mannitol.

The performance of the lyophilized and immediately reconstituted CDG-3 was evaluated and served as a zero-time point. Prior to exposure to temperature conditions, CDG-3 demonstrated slightly reduced performance post-lyophilization without excipients (Figure 1a). The combination with lactose and raffinose showed less efficient cleavage following reconstitution from the lyophilized form (Figure 1b,c), but the combination with mannitol demonstrated identical kinetics of the reaction in both lyophilized and non-lyophilized forms (Figure 1d). Following lyophilization of CDG-3, the threshold of the detection of BlaC cleavage increased from 16 nM in the non-lyophilized substrate to 81 nM. However, the correlation of fluorescence vs. BlaC concentration was close to linear in both lyophilized and non-lyophilized CDG-3 (Figure 1e,f). The fluorescent signal of the cleavage product did not differ among the non-lyophilized samples, regardless of the presence or absence of any of the excipients (Figure 1g). The overall fluorescence intensity of the product and, as a result, reduced fluorescence of the lyophilized substrate was observed (Figure 1h), but this difference was not statistically significant at the highest BlaC concentration (2022 nM). BlaC concentrations of 404 nM, 81 nM and 16 nM were lower in the lyophilized samples (*p* < 0.05, data not shown). CDG-3 with lactose and raffinose did not show statistically significant differences among the lyophilized and non-lyophilized samples at all time points, except for raffinose at 404 nM of BlaC, where it was higher in non-lyophilized samples (*p* = 0.01). Conversely, CDG-3 with mannitol demonstrated a higher fluorescent signal at 404 nM BlaC after lyophilization (*p* = 0.0046), and there were no differences at the other concentrations of BlaC (data not shown). Despite the tendency of the fluorescent signal to decrease after lyophilization, lactose and mannitol helped maintain CDG-3 function, as indicated by the levels of signal (Figure 1h). The signal was significantly higher in the presence of lactose and mannitol in lyophilized CDG-3 compared to the other two conditions, especially at low concentrations of BlaC, such as 81 nM, which is critical for the sensitivity of the test.

**Figure 2:**
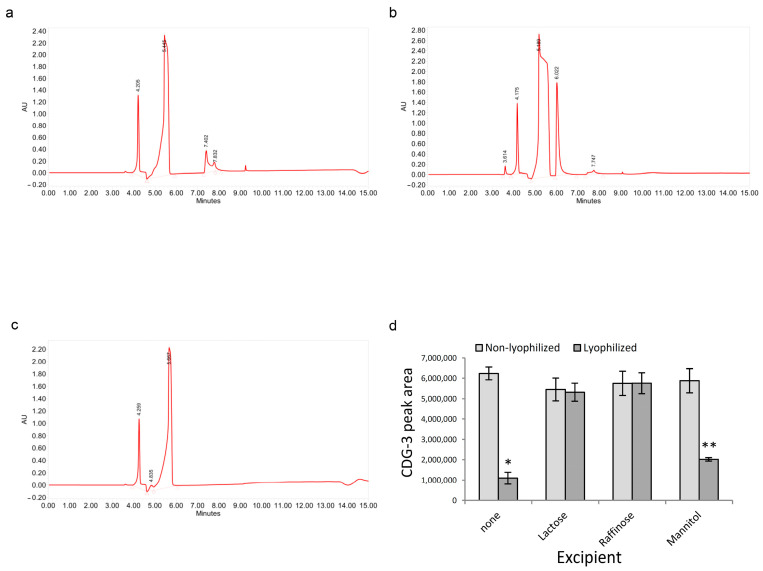
Sugar excipients helped retain CDG-3 HPLC peak area following lyophilization. CDG-3 in non-lyophilized and lyophilized forms was analyzed using HPLC. (**a**)—HPLC curve with the intact CDG-3 substrate (double peak with retention at 7.4–7.8 min); (**b**)—HPLC profile of the CDG-3 substrate after cleavage with the BlaC with the additional peak (reaction product) displaying retention at 6 min. Other peaks represent MES buffer; (**c**)—MES buffer only; (**d**)—Comparison of HPLC peaks representing CDG-3 unexposed to experimental conditions before and after lyophilization with retention at 7.5–7.7 min. Average of two experimental replicates per condition. Error bars—standard deviation. * *p* = 0.003; ** *p* = 0.011.

HPLC analysis of CDG-3 before and after BlaC cleavage showed a substrate peak retention time of 7.4–7.8 min (Figure 2). After cleavage with BlaC, the major difference was the appearance of the additional peak at 6 min, corresponding to the product of the cleavage and reduction in the substrate peak (Figure 2b).

The comparison of HPLC profiles in non-lyophilized and lyophilized samples (Figure 2d) demonstrated no change in the area of the substrate for samples lyophilized in the presence of lactose or raffinose, but they were significantly smaller in samples lyophilized with mannitol or without excipients.

### 3.2. Lyophilization of CDG-3 Substrate Helps Protect It from Degradation at High Temperature

**Figure 3:**
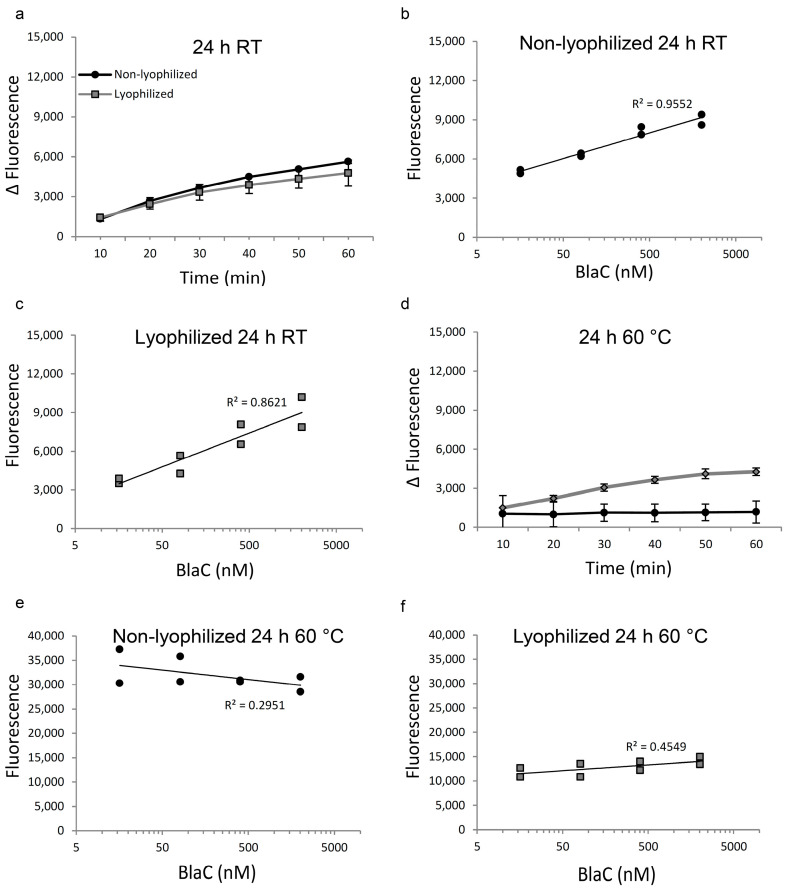
Lyophilization helps protect CDG-3 from complete degradation at high temperature. Results of the reaction of BlaC with non-lyophilized vs. lyophilized CDG-3 substrate without added excipients. (**a**)—Difference in fluorescent signal between background and CDG-3 treated with 404 nM BlaC after exposure to room temperature for 24 h; (**b**)—Relationship between BlaC concentration and fluorescent signal in non-lyophilized samples after exposure of CDG-3 to RT for 24 h; (**c**)—Relationship between BlaC concentration and fluorescent signal in lyophilized samples after exposure to RT for 24 h; (**d**)—Difference in fluorescent signal between background and CDG-3 treated with 404 nM BlaC after exposure to 60 °C for 24 h; (**e**)—Relationship between BlaC concentration and fluorescent signal in non-lyophilized samples after exposure to 60 °C for 24 h; (**f**)—Relationship between BlaC concentration and fluorescent signal in lyophilized samples after exposure to 60 °C for 24 h.

Next, we tested whether lyophilization without excipients helps preserve CDG-3 upon exposure to the high temperature. The exposure of lyophilized substrate to room temperature for 24 h demonstrated no changes in the threshold of BlaC detection, which remained at 81 nM, the same as the lyophilized substrate not exposed to room temperature for 24 h. Fluorescence intensity was reduced by 28% for the substrate exposed to RT in the lyophilized form. Exposure of liquid CDG-3 to room temperature for 24 h reduced its ability to undergo enzymatic cleavage and thus raised the threshold of BlaC detection from 16 nM to 81 nM, making it similar to the lyophilized form exposed to room temperature. A 23% reduction in fluorescence signal was observed when reacted with the BlaC at 2022 nM as compared to the lyophilized substrate not exposed to room temperature for a prolonged period. The major change in the liquid substrate was observed in the background fluorescence level. Lyophilization reduced background fluorescence by 66% as compared to the non-lyophilized substrate, all while retaining similar background to freshly produced CDG-3. There was no difference in the Δ fluorescence between lyophilized and non-lyophilized CDG-3 without excipients after exposure to RT for 24 h (Figure 3a). The correlation between the BlaC concentration and fluorescence was closer to linear in the non-lyophilized substrate than in the other conditions (Figure 3b,c).

The exposure of liquid CDG-3 to 60 °C for 24 h caused the complete inability of detection via BlaC at any concentration of the enzyme (Figure 3d). The concentration vs. the fluorescence chart showed a negative correlation (Figure 3e). Additionally, the background fluorescence of the substrate increased 11–12 times if compared to the fresh substrate, which suggested complete degradation. Lyophilized CDG-3 exposed to 60 °C for 24 h had increased background fluorescence relative to RT, but it was 3 times lower than that of non-lyophilized CDG-3 (Figure 3f). Additionally, cleavage by BlaC remained partly saved in the lyophilized form of CDG-3, as fluorescence was observed in a time-dependent and BlaC-concentration-dependent manner (Figure 3d,f).

### 3.3. Lyophilization Demonstrated the Best Protective Effect with the Presence of Lactose or Mannitol as Excipients

**Figure 4:**
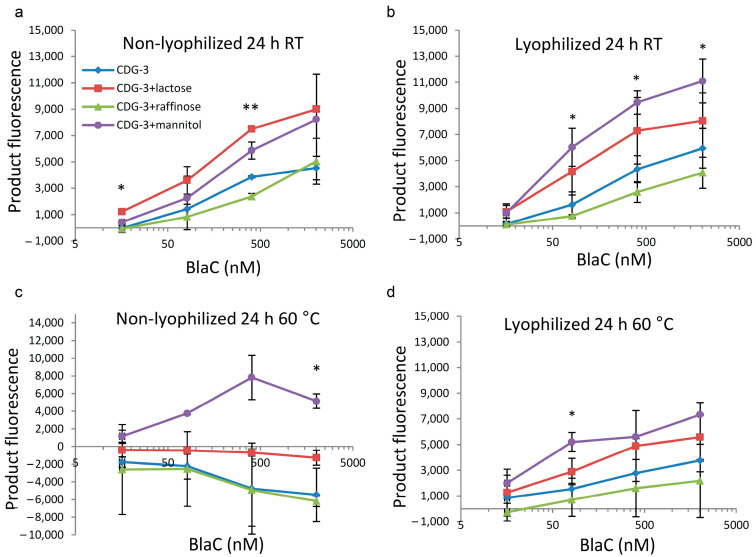
Use of lactose and mannitol as lyophilization excipients protects CDG-3 from degradation at elevated temperatures. For each data point, the difference between fluorescent signal and background fluorescence of CDG-3 is shown (30 min of exposure to BlaC). (**a**)—exposed to room temperature for 24 h in non-lyophilized form (* *p* < 0.01 in the comparison: CDG-3 vs. lactose; lactose vs. raffinose; *p* = 0.025 in the comparison of lactose vs. mannitol. ** *p* < 0.01 in the comparisons: CDG-3 vs. lactose, lactose vs. raffinose, raffinose vs. mannitol; *p* < 0.05 in: CDG-3 vs. raffinose and mannitol, lactose vs. mannitol); (**b**)—exposed to room temperature for 24 h in lyophilized form (* *p* < 0.05 in the comparison of raffinose vs. mannitol); (**c**)—exposed to 60 °C for 24 h in non-lyophilized form; * *p* = 0.01 in comparisons: CDG-3 vs. mannitol and *p* = 0.008 in the comparison of raffinose vs. mannitol); (**d**)—exposed to 60 °C for 24 h in lyophilized form (* *p* < 0.036 in the comparison of raffinose vs. mannitol).

The presence of lactose and mannitol improved the stability of lyophilized CDG-3. Interestingly, the presence of these excipients improved the results of the reaction with BlaC even without lyophilization after 24 h at RT (Figure 4a). The signal was higher in the presence of lactose or mannitol at a concentration of 404 nM BlaC and only lactose at 16 nM BlaC. There was also an increase in the fluorescent signal of the lyophilized samples in the presence of mannitol or lactose, but a statistically significant difference was only observed between mannitol and raffinose at 2022, 404 and 81 nM BlaC (Figure 4b).

After incubation at 60 °C as a liquid, the product of CDG-3 cleavage had lower fluorescence that the substrate fluorescence background in all combinations, except CDG-3 + mannitol (Figure 4c). The samples with mannitol showed positive average values with significantly higher levels in the samples with mannitol vs. CDG-3 only and raffinose vs. mannitol for the highest concentration of BlaC. Nevertheless, lyophilized samples still demonstrated positive product fluorescence and positive changes in fluorescent signal intensity along with increasing BlaC concentrations. CDG-3 with mannitol showed significantly higher fluorescence than with raffinose at 81 µM of BlaC (Figure 4d).

**Figure 5:**
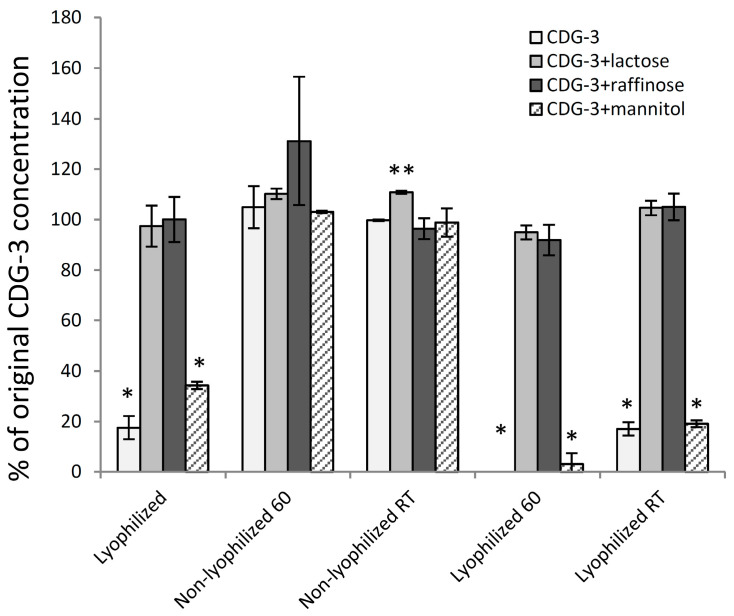
Lyophilization excipients demonstrate differing protection from high temperature. Result of HPLC analysis of the substrate after lyophilization and exposure to various experimental conditions shown. Ratios of HPLC peak areas of the substrate after exposure to experimental conditions and unexposed substrate were determined. These data suggest that combinations of excipient sugars may further improve CDG-3 reagent stability at elevated temperature following reconstitution. Two experimental replicates for each condition. Error bars represent the standard deviation. * different from lactose and raffinose (*p* < 0.01); ** different from raffinose (*p* < 0.05).

HPLC analysis of CDG-3 exposed to various experimental conditions was performed, and the area of the CDG-3 peak was measured and presented in Figure 5 as a percentage of the area of the original, non-lyophilized CDG-3. The comparison of the effect from each sugar at each experimental condition demonstrated that the highest level of preservation of the CDG-3 area occurred with lactose and raffinose in all lyophilized samples, plus all non-lyophilized combinations. Non-lyophilized samples exposed to 60 °C demonstrated full preservation of the CDG-3 area without excipients and with all tested sugars, according to the HPLC analysis, suggesting that temperature incubation does not change HPLC mobility or concentration of CDG-3.

### 3.4. Lyophilization Did Not Affect Albumin Absorption by Blue Sepharose

The presence of albumin in the samples may have adverse effects on CDG-3 cleavage by BlaC, and as such, it is necessary to remove albumin that can be present in a clinical specimen, such as sputum. Blue Sepharose serves as an albumin removal agent. It is important to validate Blue Sepharose’s ability to survive the freeze-dry procedure and reconstitution, as well as resistance to harsh conditions when in the lyophilized form.

Five cycles of albumin extraction (1 h on a shaker at room temperature) were performed, replacing Blue Sepharose after each cycle and sampling the buffer for albumin concentration.

**Figure 6:**
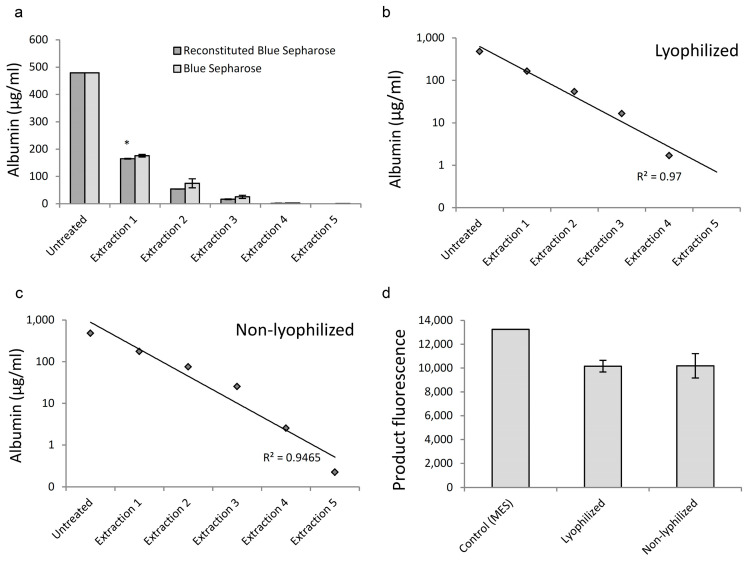
Ability of Blue Sepharose to deplete albumin is not reduced by lyophilization. (**a**)—Decrease in albumin concentration after five cycles of extraction with lyophilized and non-lyophilized Blue Sepharose, *n* = 2, error bars = standard deviation, * *p* = 0.046; (**b**,**c**)—Linear relationship between albumin concentration and extractions; (**d**)—Albumin depleted buffer was used in the cleavage of CDG-3 by BlaC (404 nM). *n* = 2 (experimental replicates), error bars = standard deviation.

Lyophilized and non-lyophilized Blue Sepharose was tested in parallel. As a result, lyophilized and reconstituted Blue Sepharose demonstrated even slightly better albumin removal ability, as the albumin concentration was lower after every cycle of extraction. After the first cycle, this difference was statistically significant (Figure 6a). Approximately one-third of the previous concentration of albumin remained with each extraction cycle, and after the fifth cycle of lyophilized Blue Sepharose treatment, albumin was undetectable. Meanwhile, non-lyophilized Blue Sepharose treated samples had 0.04% of the original concentration of albumin. The reduction in albumin concentration was very close to linear with each extraction when treated with both lyophilized and non-lyophilized Blue Sepharose (Figure 6b,c).

After five cycles of albumin depletion, the buffer was tested in the cleavage reaction of CDG-3 with BlaC (Figure 6d). Differences in the signal/background ratio within 30 min of incubation were not observed among both experimental conditions.

**Figure 7:**
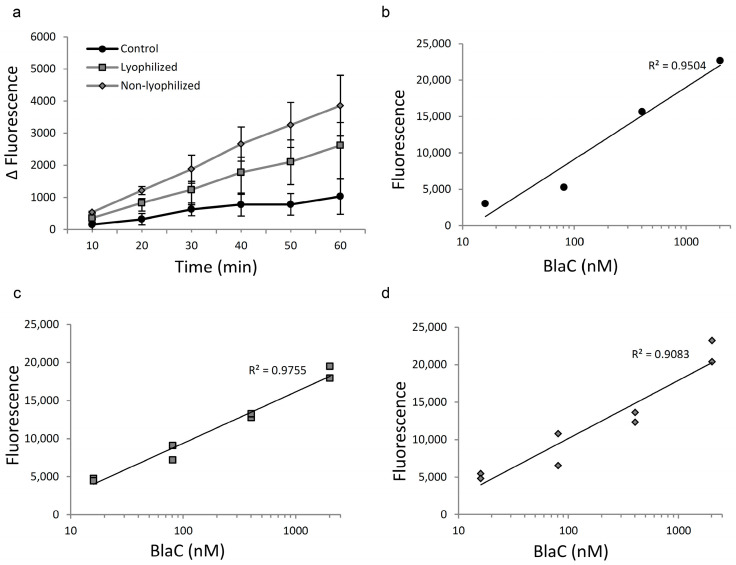
Albumin depletion of REFtb reaction buffer with Blue Sepharose significantly improves fluorescence levels compared to the buffer without albumin and Blue Sehparose. Comparison of the fluorescent signal in the product of CDG-3 cleavage using buffers after five cycles of albumin extraction with lyophilized and non-lyophilized Blue Sepharose. Albumin-free buffer served as a control. (**a**)—Kinetics of the reaction with 16 um BlaC and CDG-3 substrate; (**b**)—Linear relationship between BlaC concentration and fluorescence of the product of CDG-3 cleavage in the control; (**c**)—buffer treated with lyophilized Blue Sepharose and (**d**)—buffer treated with non-lyophilized Blue Sepharose.

Interestingly, the reaction kinetics demonstrated improvement in both buffers treated with lyophilized and non-lyophilized Blue Sepharose (Figure 7a) when compared with albumin-free buffer, especially at lower BlaC concentrations. This was confirmed by an improved linear relationship between the BlaC and fluorescent signal intensity in the MES buffer that was treated with lyophilized Blue Sepharose (Figure 7c) when compared with albumin-free buffer (Figure 7b) and treated with non-lyophilized Blue Sepharose (Figure 7d).

### 3.5. Exposure of Blue Sepharose to Higher Temperatures Did Not Affect Its Ability to Absorb Albumin

The microscopy of lyophilized and then reconstituted Blue Sepharose samples demonstrated no visual changes. The beads were visually identical to fresh non-lyophilized beads. Blue Sepharose that was exposed to higher temperatures, including 60 °C for 24 h, in both liquid and lyophilized forms, was microscopically identical to the fresh sample. There was no damage, deformation, discoloration or any other changes observed (not shown).

**Figure 8:**
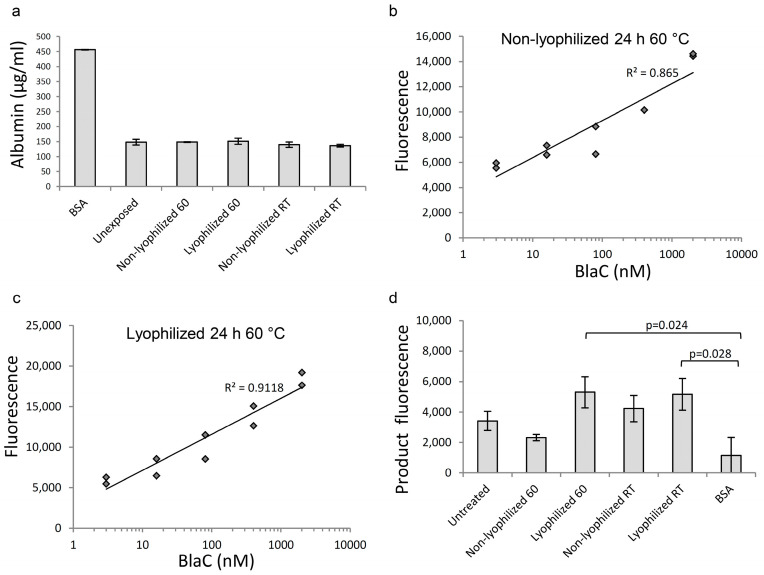
Blue Sepharose retains the ability to deplete albumin following lyophilization and exposure to adverse temperatures. Non-lyophilized Blue Sepharose was exposed to RT and 60 °C for 24 h and tested for the ability to bind albumin. (**a**)—effect of temperature on the albumin-binding ability of Blue Sepharose in lyophilized and non-lyophilized forms, two experimental replicates, error bars = standard deviation; (**b**)—The relationship of BlaC concentration and fluorescent signal in the buffer spiked with albumin and treated with Blue Sepharose that was exposed 24 h to 60 °C in non-lyophilized form; (**c**)—The relationship of BlaC concentration and fluorescent signal in the buffer spiked with albumin and treated with Blue Sepharose that was exposed 24 h to 60 °C in lyophilized form; (**d**)—Fluorescence of CDG-3 cleavage product with subtracted background signal in albumin-spiked buffers, treated with lyophilized and non-lyophilized Blue Sepharose (30 min of BlaC treatment, 404 nM BlaC). Only two comparisons (shown on the panel) are significantly different. Two replicates.

The exposure of Blue Sepharose to the higher temperatures in both the lyophilized and non-lyophilized forms, after resuspension in the MES buffer, did not affect its ability to bind albumin (Figure 8a). One cycle of treatment with Blue Sepharose removed 67% of albumin in all samples, regardless of the temperature of exposure or lyophilization status. Buffers after treatment with Blue Sepharose were used to evaluate the effect of treatment on their ability to maintain CDG-3 cleavage via purified BlaC. As a result, the linear relationship between BlaC concentration and fluorescence intensity was improved in the buffer treated with lyophilized Blue Sepharose exposed to 60 °C compared to non-lyophilized Blue Sepharose in the same conditions (Figure 8b,c). The same difference was observed between lyophilized and non-lyophilized samples at room temperature (data not shown). The fluorescence of the product (Figure 8d) was not different among buffers exposed to any experimental conditions. The fluorescence of the product was higher than the BSA control in buffers treated with lyophilized Blue Sepharose. All experimental conditions had no effect and were not significantly different from the intact MES buffer that was not spiked with BSA.

## 4. Discussion

Lyophilization includes a series of procedures, such as freezing the material prior to placing it under vacuum to sublimate the water [36]. This process helps preserve biological and pharmaceutical materials [35]; however, it is important to ensure the process used does not have an adverse effect on the compounds being preserved. In this study, we demonstrated that freezing, lyophilization and reconstitution of the lyophilized form of CDG-3 in the MES buffer does not lead to degradation. Overall, lyophilization did not affect fluorescence intensity of the product of CDG-3 cleavage by BlaC nor the change in fluorescence. However, adding lactose as an excipient helped improve the performance of the lyophilized substrate even further, as observed by an increase in fluorescent signal, returning the ability to detect cleavage of the substrate by BlaC to the level of non-lyophilized CDG-3. Lactose is a disaccharide, which includes D-galactose and D-glucose units linked through a β (1–4) glycosidic bond. Lactose is mainly used as a soluble diluent and binding agent [37]. Two anomeric forms of lactose, α and β, exist. They differ in physical properties, such as the melting point, specific optical rotation, density and solubility [37]. For this study, we selected α-lactose monohydrate, as it is approximately 10 times less expensive than β-lactose, according to our goal of keeping the price of the REF test as low as possible. Upon lyophilization, this composition formed a porous cake that was rapidly reconstituted (within 60 s). The most important result observed using lactose as an excipient was the stabilization of CDG-3 and a reliable performance under all experimental conditions tested. In the presence of lactose, CDG-3 demonstrated the best stability in both BlaC detection and HPLC analyses.

Mannitol displays significant utility in protecting CDG-3 from degradation when exposed to high temperature, as would be experienced during non-refrigerated transport. However, more CDG-3 was lost during lyophilization than when using other excipients. We found a direct connection between temperature and the level of background fluorescence of CDG-3. The exposure to high temperature for prolonged time likely caused structural changes in CDG-3, preventing product cleavage and subsequent fluorescence. Overall, in this study, both lactose and mannitol showed the ability to retain excellent CDG-3 activity after lyophilization, even with treatment at high temperature. The combination of activity retention and efficient lyophilization and reconstitution make lactose the best excipient that we tested in this study, which is a result similar to other studies, such as the one where excipients were used for lyophilization of methylprednisone. The authors demonstrated that the rate of methylprednisone hydrolysis was significantly higher in formulations containing mannitol vs. lactose due to the crystallization of mannitol. The physical state in lactose remained constant, and the rate of hydrolysis was not significantly different from the control formulation [38]. Since CDG-3 cleavage involves the hydrolysis of the lactam ring [33], it is possible that a similar mechanism of CDG-3 degradation takes place when we expose it to higher temperatures, even in the lyophilized form with mannitol as an excipient.

When the CDG-3 substrate was exposed to room temperature or 60 °C, lyophilization helped preserve it even without excipients, as evidenced by the level of background fluorescence being consistently lower in the lyophilized samples. Interestingly, our HPLC analysis indicated that lyophilization, in the absence of excipients, leads to a reduction in detectability of CDG-3. This suggests that one important role of the excipient may be to allow for the stability of active ingredients at low concentrations. Despite the apparent loss of CDG-3, the substrate retained functional activity in all the lyophilized samples, even after exposure to 60 °C. CDG-3 can be cleaved and detected at the same concentration of BlaC as freshly made substrate. The only observed difference was higher background fluorescence. Therefore, lyophilization is promising for CDG-3 preservation and, when combined with the right excipient, demonstrates higher stability even under relatively harsh conditions.

Another component of the REFtb reagents is Blue Sepharose, which consists of Cibacron Blue, immobilized on agarose beads. Blue Sepharose is well known for its human serum albumin absorption [39] and is widely used in affinity chromatography and purification of proteins [40]. The albumin-binding property of Blue Sepharose allows depletion of albumin in clinical specimens of sputum. It is well known that sputum contains albumin, but the concentration may vary depending on the patient [41]. In this study, we validated lyophilization as having no obvious adverse effects on its structural integrity or albumin-binding ability. Both lyophilized and non-lyophilized Blue Sepharose depleted albumin in solution with the same efficiency. The MES buffer with albumin that was then treated with Blue Sepharose allowed the same levels of CDG-3 cleavage by BlaC for both the previously lyophilized and non-lyophilized versions of Blue Sepharose. Interestingly, the threshold of BlaC detection was much lower in the treated buffers than in the untreated control buffer (without albumin). It is possible that the presence of trace amounts of albumin is beneficial for optimal BlaC function. Another explanation is that the prolonged (60 min) treatment of the MES buffer with Blue Sepharose had a conditioning effect on the buffer by sequestering an unknown inhibitor.

High-temperature treatment had no adverse effect on Blue Sepharose protein binding ability. Incubation at room temperature and 60 °C in the lyophilized form and in the buffer had no obvious effect on the performance of Blue Sepharose. The remaining concentration of albumin after treatment was the same, regardless of the temperature for Blue Sepharose. The highest CDG-3 cleavage by BlaC was observed when albumin-spiked buffer was treated with Blue Sepharose that was exposed to 60 °C for 24 h in the lyophilized form. CDG-3 cleavage in this buffer caused the highest fluorescent signal of the product. Based on this observation, we should consider the introduction of heat treatment of Blue Sepharose as part of the reagent preparation protocol for REFtb.

Overall, in this work, we found that we could lyophilize the substrate and reagents for REFtb without compromising the performance as compared to the current liquid-based diagnostic if excipients were present. Good stability of the reagents was maintained, even with high-temperature treatment for 24 h. Based on the data from reconstitution as well as maintenance of the substrate concentrations and activity at multiple temperatures, lactose and mannitol displayed better performance overall than raffinose as excipients.

The major direction of further research should be optimization of the lyophilization protocol, including adjustments in excipient concentration and possible combinations of lactose and mannitol. Considering the fact that the enzymatic reaction in this study was performed in buffer, it is conceivable that sputum will impact aspects of reconstitution or enzyme activity, but our prior studies suggest that there is little difference between data obtained in buffer and sputum [26]. Ongoing studies will examine the long-term and/or accelerated stability testing of REFtb reagents along with additional validation in clinical samples from diverse geographic areas. Interestingly, based on our data, heat pre-treatment of Blue Sepharose may increase the ability of Blue Sepharose to remove albumin, suggesting that pre-treatment is also something that should be examined as a strategy to improve REFtb performance. These studies, therefore, provided valuable insight into the best approach for lyophilization to stabilize the REFtb diagnostic system for shipping and long-term storage, which is an important step toward the implementation of REFtb testing in the field.

## 5. Conclusions

Summarizing the results of this study, we can draw the following conclusions:

(1) Lyophilization procedures had minimal negative effects on the function of the CDG-3 substrate, but the use of lactose and mannitol as excipients helped enhance the stability of lyophilized CDG-3.
(2) Lyophilization helped protect the CDG-3 substrate from the damaging effects of high temperature.
(3) The presence of lactose or mannitol enhanced the protective effects of lyophilization against high temperatures.
(4) Blue Sepharose did not lose its ability to absorb albumin after lyophilization and re-constitution.
(5) Exposure to high temperatures did not reduce the ability of Blue Sepharose to absorb albumin.

## Data Availability

Not applicable.
